# Lipopolysaccharide (LPS)-induced inflammation in RAW264.7 cells is inhibited by microRNA-494-3p via targeting lipoprotein-associated phospholipase A2

**DOI:** 10.1007/s00068-024-02588-7

**Published:** 2024-07-02

**Authors:** Wenxiao Yan, Yan Yan, Xinye Luo, Yansong Dong, Guiwen Liang, Hua Miao, Zhongwei Huang, Haiyan Jiang

**Affiliations:** 1 Department of Emergency Medicine, Affiliated Hospital of Nantong University, Nantong, China; 2 Medical School of Nantong University, Nantong University, Nantong, China; 3 Department of Intensive Care Unit, Second Affiliated Hospital of Jiaxing University, Jiaxing, China; 4 Department of Emergency Medicine, Rudong County People’s Hospital, Nantong, China

**Keywords:** Sepsis, microRNAs, miR-494-3p, Lipopolysaccharide, Lipoprotein-associated phospholipase A2

## Abstract

**Background:**

Gram-negative bacterial lipopolysaccharide (LPS) is a major component of inflammation and plays a key role in the pathogenesis of sepsis. According to our previous study, the expression of lipoprotein-associated phospholipase A2 (Lp-PLA2) is significantly upregulated in septic patients and is positively correlated with the severity of this disease. Herein, we investigated the potential roles of Lp-PLA2-targeting microRNAs (miRNAs) in LPS-induced inflammation in murine mononuclear macrophages (RAW264.7 cells).

**Methods:**

In LPS-stimulated RAW264.7 cells, Lp-PLA2 was confirmed to be expressed during the inflammatory response. The function of microRNA-494-3p (miR-494-3p) in the LPS-induced inflammatory response of RAW264.7 cells was determined by the transfection of a miR-494-3p mimic or inhibitor in vitro.

**Results:**

Compared to the control, LPS induced a significant increase in the Lp-PLA2 level, which was accompanied by the release of inflammatory mediators. The bioinformatics and qRT‒PCR results indicated that the miR-494-3p level was associated with Lp-PLA2 expression in the LPS-induced inflammatory response of RAW264.7 cells. Dual-luciferase reporter assay results confirmed that the 3’-UTR of Lp-PLA2 was a functional target of microRNA-494-3p. During the LPS-induced inflammatory response of RAW264.7 cells, targeting Lp-PLA2 and transfecting miR-494-3p mimics significantly upregulated the expression of miR-494-3p, leading to a reduction in the release of inflammatory factors and conferring a protective effect on LPS-stimulated RAW264.7 cells.

**Conclusion:**

By targeting Lp-PLA2, miR-494-3p suppresses Lp-PLA2 secretion, thereby alleviating LPS-induced inflammation, which indicates that miR-494-3p may be a potential target for sepsis treatment.

## Introduction

Sepsis, described as an uncontrolled inflammatory response that results in life-threatening organ damage, has emerged as an important global health issue [1]. According to global burden of disease studies, the incidence of sepsis decreased from 1990 to 2017, but alarmingly, 19.7% of all deaths in 2017 were related to sepsis [2]. Although prevention and treatment strategies have improved significantly, sepsis remains one of the leading causes of death among critically ill patients worldwide [2]. In gram-negative bacterial infections, lipopolysaccharides (LPS), which are key components of the pathogenesis of sepsis, are released. In sepsis, mononuclear macrophages play an important role as members of the innate immune system [3]. Inflammatory cytokines, such as TNF-α, IL-1β, and IL-6, are released by mononuclear macrophages as part of their function [4]. Sepsis-associated multiple organ failure can occur as a result of the excessive release of inflammatory cytokines [5, 6]. It is therefore clear that the activation of mononuclear macrophages plays an important role in the inflammatory response and multiorgan failure triggered by sepsis [7]. It is highly important to investigate the activation and regulatory mechanisms of monocyte macrophages in sepsis.

MicroRNAs (miRNAs) are a class of short noncoding RNAs that bind to the 3’-untranslated region (UTR) of target mRNAs, degrading those target mRNAs and inhibiting their translation [8, 9]. Several miRNAs have been shown to be associated with LPS-induced macrophage inflammation [10–13]. However, the functions of many miRNAs in inflammatory responses have yet to be explored.

Lipoprotein-associated phospholipase A2 (Lp-PLA2), a unique member of the PLA2 family, is also known as platelet-activating factor acetylhydrolase (PAF-AH) [14, 15]; it is an inflammatory factor that has been widely studied in recent years. Lp-PLA2 is primarily secreted by mononuclear macrophages [16], and after its release, it binds partially to low-density lipoprotein B, which is then oxidized and cleaved into bioactive compounds, such as oxidized phosphatidylcholine, lysophosphatidylcholine, and oxidized free fatty acids, all of which are believed to have inflammation-inducing properties [16, 17]. Our previous studies have shown that Lp-PLA2 is highly expressed in patients with sepsis and is positively correlated with disease severity. Therefore, Lp-PLA2 has prognostic value in the early diagnosis of sepsis and evaluation of prognosis [18].

## Methods

### Cell culture and stimulation

Murine macrophages (RAW 264.7 cells) were purchased from the Cell Bank of the Chinese Academy of Sciences (Shanghai, China) and grown in Dulbecco’s modified Eagle’s medium (DMEM, Gibco, USA) supplemented with 10% foetal bovine serum (FBS, Gibco, USA), 100 U/mL penicillin, and 100 µg/mL streptomycin (C100C5, New Cell and Molecular Biotech, China) in a humidified atmosphere with 5% CO_2_ at 37 °C. At a dose of 1 µg/mL, LPS (Sigma, USA) was used to establish the septic cellular model. Additionally, parallel cultures of control cells were generated without treatment. A sample of cells or supernatants was collected for analysis at the appropriate time points.

### Cell transfection

The miR-494-3p mimic (5′-UGAAACAUACACGGGAAACCUC-3′; 50 nM), mimic negative control (mimic NC, 5′-UUUGUACUACACAAAAGUACUG-3′; 50 nM), miR-494-3p inhibitor (5′-ACUUUGUAUGUGCCCUUUGGAG-3′; 50 nM), and inhibitor NC (5′-CAGUACUUUUGUGUAGUACAAA-3′; 50 nM) were synthesized, and those oligonucleotides were transfected using a RiboFECT^™^ CP Transfection Kit (all from RiboBio, Guangzhou, China) in accordance with the manufacturer’s instructions. After 24 h of transfection, cells in 6-well culture plates (1.2 × 10^6^ cells/well) were stimulated with 1 µg/ml LPS at 37 °C in 5% CO_2_ for 24 h. The expression levels of miR-494-3p, Lp-PLA2, TNF-α, IL‐6 and IL‐1β were evaluated by qRT‒PCR and ELISAs.

### Cell proliferation assay

The RNA mimic, mimic NC, inhibitor and inhibitor NC were transfected into 30–50% confluent RAW 264.7 cells. The cells were resuspended for two days and then seeded into 96-well culture plates (5000 cells/well) overnight. Complete culture medium containing LPS (1 µg/mL) was added, and the cells were incubated for 24 h. Then, 10 µL of CCK-8 solution (C0039, Beyotime, Shanghai, China) was added to each well, followed by incubation at 37 °C and 5% CO_2_ for 1.5 h. The optical density at a wavelength of 450 nm (OD450) was measured. Cell proliferation was analysed by EdU staining (C0071S, BeyoClick™ EdU Cell Proliferation Kit with Alexa Fluor 488, Beyotime, Shanghai, China). We incubated the cells with EdU for 2 h, fixed the cells with 4% paraformaldehyde for 30 min, and permeabilized the cells with 0.3% Triton X-100 for another 30 min. The cells were incubated with the Click Reaction Mixture for 30 min at room temperature in the dark before being incubated with Hoechst 33,342 for 10 min.

### Determination of nitric oxide (NO) content

The supernatants from each well were collected, and the relative content of nitric oxide (NO) in the supernatants was measured. The relative NO content was measured with a NO assay kit (S0021, Beyotime Institute of Biotechnology, Shanghai, China) according to the instructions provided by the manufacturer. Each experiment was independently performed three times.

### Measurement of acid phosphatase (ACP) activity

Acid phosphatase (ACP) activity was measured with a commercially available acid phosphatase assay kit (P0326, Beyotime, Shanghai, China) according to the manufacturer’s instructions. After treatment using the aforementioned method in 6-well culture plates, lysed cells were harvested, and the supernatant was collected. The absorbance of each well at 405 nm was quantified.

### Enzyme-linked immunosorbent assays (ELISAs)

ELISA kits for the detection of Lp-PLA2 (CSB-E08321m, Cusabio, USA), TNF-α (SEKM-0034, Solarbio, China), IL-6 (SEKM-0007, Solarbio, China) and IL-1β (SEKM-0002, Solarbio, China) were obtained from Solarbio (Solarbio, Beijing, China). The levels of Lp-PLA2, TNF-α, IL-6 and IL-1β were analysed with corresponding ELISA kits according to the manufacturer’s instructions. Then, the absorbance of the coloured product was measured at 405 nm by an automated plate reading system (FlexStation 3, Molecular Device, Sunnyvale, CA, USA). Each experiment was independently performed three times.

### Dual-luciferase reporter assays

The 3′-UTR sequence of Lp-PLA2 was cloned and inserted into the pmiR-RB-Report^™^ reporter plasmid to verify that miR-494-3p can bind to the miR-494-3p target Lp-PLA2. With respect to the pmiR-RB-Report^™^ vector (RiboBio, Guangzhou, China), the wild-type and mutant 3′-UTR fragments were cloned downstream of the luciferase reporter gene. Before transfection, RAW 264.7 cells were seeded into 96-well plates at a density of 1 × 10^4^ cells/well. Then, the cells were cotransfected with the luciferase reporter vectors and the miR-494-3p mimic or the corresponding negative control using a RiboFECT^™^ CP Transfection Kit. After 24 h, luciferase activity was analysed using the Dual-Luciferase® Reporter Assay system (Promega Corporation). The relative changes in luciferase activity via *Renilla* fluorescence were calculated to identify the regulatory effect of miRNAs on this gene.

### RNA isolation, reverse transcription‒PCR and real-time quantitative PCR (qRT‒PCR)

Total RNA was isolated from cells by using RNAiso Plus (Takara, Beijing, China) and dissolved in RNase-free water according to the manufacturer’s instructions. Quality inspection and control were conducted using a NanoDrop spectrophotometer (Thermo Fisher Scientific, USA) with optical density measurements at A260/A280 = 1.8 − 2.2. Reverse transcription was performed using HiScript® II Q RT SuperMix for qRT‒PCR (+ gDNA wiper) (Vazyme, Nanjing, China). Then, 1 µg of total RNA, RNase-free ddH_2_O and 4× gDNA wiper mix were incubated at 42 °C for 2 min to remove genome contamination, and 5× HiScript II qRT SuperMix II was added to the reaction mixture. The mixture was gently blown and mixed well and then incubated at 50 °C for 15 min and 85 °C for 5 s. A LightCycler® Carousel-Based System apparatus (Roche Applied Science, Mannheim, Germany) with primers for specific mRNAs, ddH_2_O and ChamQ Universal SYBR qRT‒PCR Master Mix (Vazyme, Nanjing, China) was used for real-time PCR of the resulting cDNA. The mixtures were quantified using a real-time device under standard thermal conditions: 95 °C for 30 s; 40 cycles of 95 °C for 10 s and 60 °C for 30 s; followed by 95 °C for 15 s, 60 °C for 30 s and 95 °C for 15 s. All results were normalized to GAPDH. The 2^-ΔΔCq^ method was used to quantify the relative gene expression levels. As shown in Table 1, the sequences of the primers for GAPDH, IL-1β, IL-6 and TNF-α were designed with the National Center for Biotechnology Information Primer-Blast tool and synthesized by Genewiz, Inc. (Genewiz, Suzhou, China). Three independent experiments were performed for each reaction in triplicate.

With U6 small nuclear RNA as an internal reference, miR-494-3p levels were quantified by qRT‒PCR. Bulge-Loop™ miRNA primer sets (one RT primer and a pair of qRT‒PCR primers for each set) specific for miR-494-3p were designed by RiboBio (Guangzhou, China). The sequences of the miR-494-3p RT primer (ssD809230229), miR reverse primer (ssD089261711), and 494-3p forward primer (ssD809230921) are proprietary.

**Table 1:** Primer sequences for qRT‒PCR

Gene	Forward primer (5′-3′)	Reverse primer (5′-3′)
*TNF-a*	TATGGCTCAGGGTCCAACTC	GGAAAGCCCATTTGAGTCCT
*IL-1β*	AGTTGACGGACCCCAAAA	AGCTGGATGCTCTCATCAGG
*IL-6*	CACGGCCTTCCCTACTTCAC	TGCAAGTGCATCATCGTTGT
*GAPDH*	CAGGTTGTCTCCTGCGACTT	TATGGGGGTCTGGGATGGAA

GAPDH, glyceraldehyde-3-phosphate dehydrogenase

### Statistical analysis

All experiments were repeated at least three times. Statistical calculations were performed using IBM SPSS Statistics 20 and GraphPad Prism 8, and the data are presented as the mean ± standard deviation (SD). Differences between groups were tested using Student’s t test for two groups and one-way ANOVA or two-way ANOVA for three or more groups. Differences between groups were considered significant at *p* < 0.05.

## Results

### Expression of Lp-PLA2 in the inflammatory response of LPS-stimulated RAW264.7 cells

**Fig. 1:**
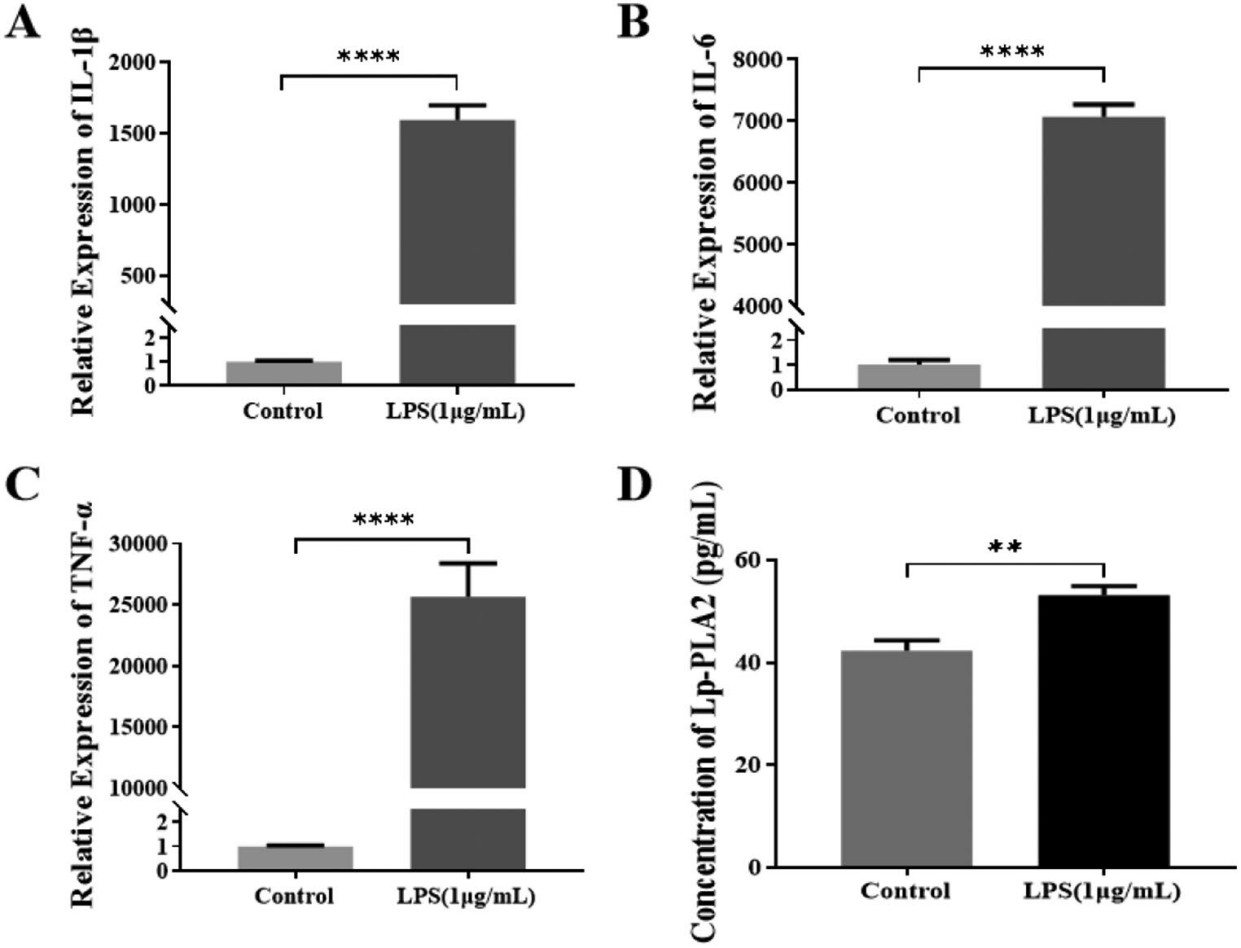
Relative expressions and concentrations of IL-1β, IL-6, TNF-α, and Lp-PLA2. (**A**, **B**, and **C**) The mRNA expression levels of IL-1β, IL-6, and TNF-α in RAW264.7 cells 24 h after LPS (1 µg/mL) stimulation as measured by qRT‒PCR. (**D**) The concentrations of Lp-PLA2 as measured by ELISA. Parametric data were analysed by paired t tests and are displayed as the mean ± SD. *N* = 3, ** *p* < 0.01, **** *p* < 0.0001

As part of our preliminary study, we conducted a statistical analysis of the serum levels of Lp-PLA2, C-reactive protein, procalcitonin, and interleukin 6, sequential organ failure (SOFA) scores and acute physiology and chronic health evaluation II (APACHE II) scores between 151 patients with sepsis (39 with sepsis, 55 with severe sepsis and 57 with septic shock) and 30 healthy controls. According to our findings, Lp-PLA2 levels were significantly greater in sepsis patients than healthy control subjects. Furthermore, we observed a positive correlation between Lp-PLA2 levels and APACHE II scores as well as mortality rates. In addition, Lp-PLA2 was positively associated with commonly used clinical inflammatory markers such as CRP, PCT, and IL-6. Therefore, we believe that Lp-PLA2 can be used as an indicator for assessing the severity and prognosis of sepsis in a clinical setting [18]. To further understand the role of Lp-PLA2 in sepsis pathogenesis, we conducted in vitro experiments. In the initial studies, we measured the secretion of inflammatory cytokines from LPS-stimulated RAW264.7 cells using qRT‒PCR and found that the levels of IL-1β, IL-6, and TNF-α were significantly higher in LPS-stimulated cells than in control cells (Fig. 1A-C). Subsequently, ELISAs were employed to quantify the concentration of Lp-PLA2 in cell culture supernatants, revealing significantly elevated levels in the LPS group compared to those in the control group (Fig. 1D). Consequently, we conclude that inflammation induced by LPS increases the secretion of Lp-PLA2.

### Lp-PLA2 is targeted by miR-494-3p

**Fig. 2:**
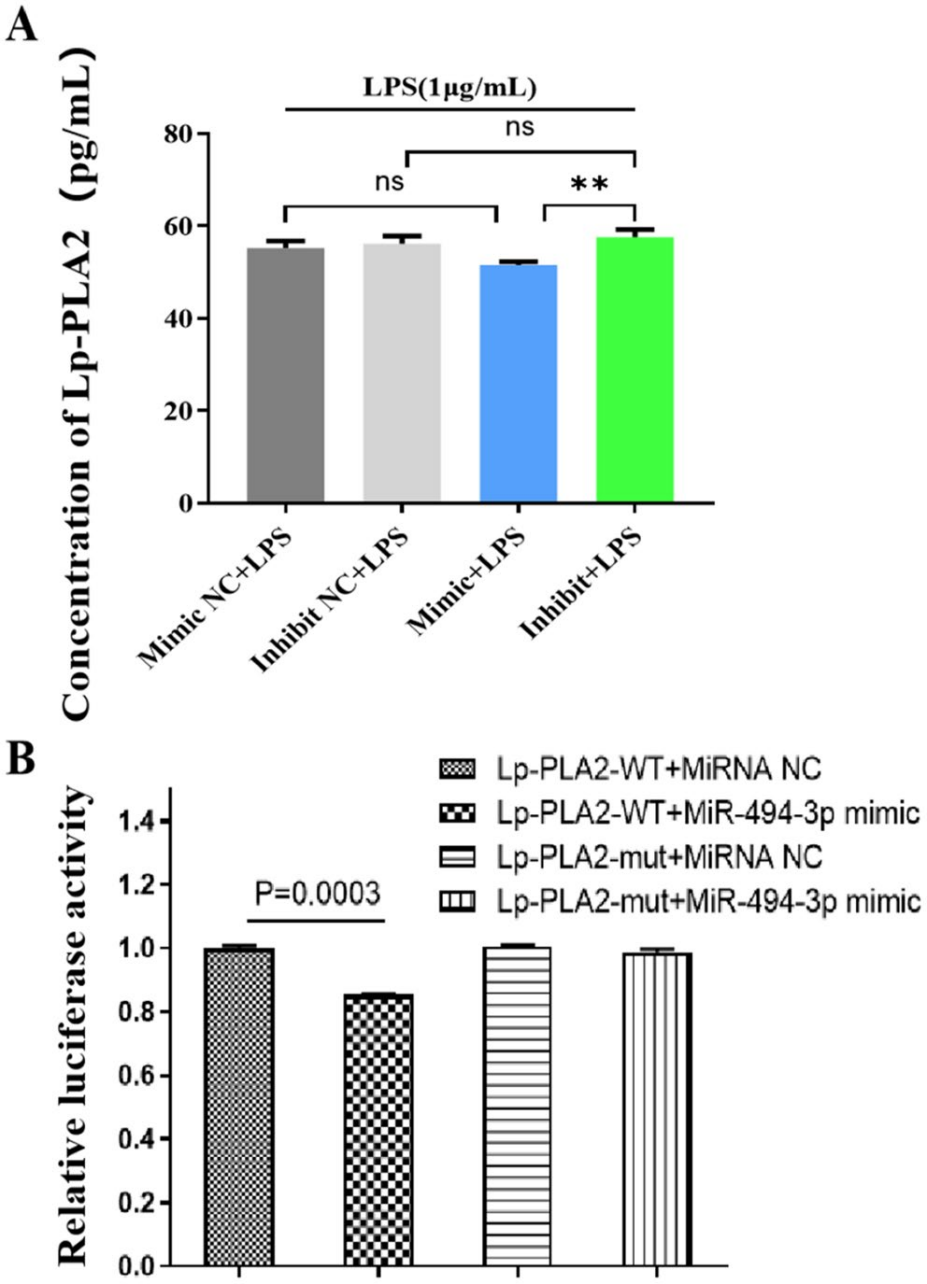
Cell proliferation and luciferase assay results. (**A**) The concentrations of Lp-PLA2 as determined by ELISA. (**B**) The results of the luciferase assay showed the relative activity of Lp-PLA2, indicating that miR-494-3p mimic transfection significantly inhibited wild-type luciferase activity in the 3′-UTR of Lp-PLA2 but had no effect on the mutated 3′-UTR of Lp-PLA2 (*p* < 0.05). Parametric data were analysed by paired t tests and are displayed as the mean ± SD. *N* = 3, * *p* < 0.05, ** *p* < 0.01, *** *p* < 0.001

Using bioinformatics databases such as TargetScan, miRanda, and PicTar, we screened for miRNAs that may modulate the expression of Lp-PLA2 to identify potential regulatory molecules. Through a comprehensive analysis of the literature, we were able to establish a strong correlation between miR-494-3p and Lp-PLA2. To investigate the cellular secretion of Lp-PLA2, we transfected a miR-494-3p mimic and inhibitor into RAW264.7 cells. Based on the results, the miR-494-3p mimic significantly suppressed Lp-PLA2 release in RAW264.7 cells, whereas the miR-494-3p inhibitor enhanced Lp-PLA2 release, suggesting that miR-494-3p has an inhibitory effect on Lp-PLA2 (Fig. 2A). Furthermore, we performed dual-luciferase reporter assays to further validate the correlation. Using the pmiR-RB-Report^™^ vector, we cloned the predicted target sequences of miR-494-3p from the 3’-UTR of Lp-PLA2 and mutants. Then, we cotransfected the miR-494-3p mimic and the pmiR-RB-Report^™^ vector into RAW264.7 cells. Inhibition of luciferase activity was observed following transfection with the miR-494-3p mimic and pmiR-RB-Report^TM^–Lp-PLA2-wild-type plasmid but not with the pmiR-RB-Report^TM^–Lp-PLA2-mutant plasmid, indicating that miR-494-3p binds specifically to the 3′-UTR of Lp-PLA2.

### Role of mir-494-3p in the inflammatory response of LPS-stimulated in RAW264.7 cells

**Fig. 3:**
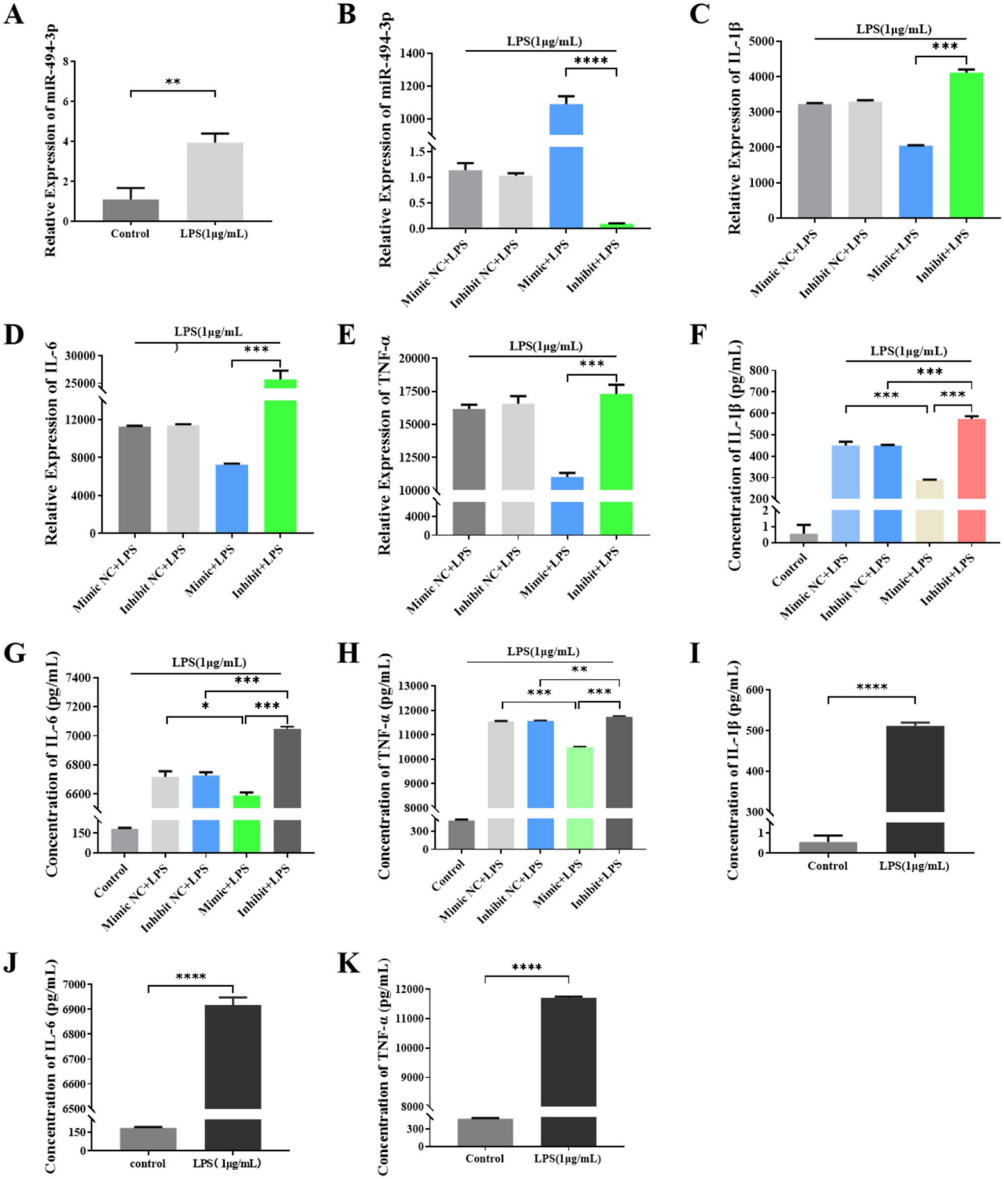
Expression of inflammatory factors in RAW264.7 cell culture supernatants was affected by transfection with miR-494-3p. (**A**) qRT‒PCR was used to determine the relative expression of miR-494-3p in RAW264.7 cells 24 h after LPS stimulation (1 µg/mL). (**B**) RAW264.7 cells were transfected with 50 nM miR-494-3p mimic, inhibitor or NC. Transfection efficiency was confirmed 24 h later by qRT‒PCR later. (**C**, **D** and **E**) Expression levels of IL-1β, IL-6 and TNF-α mRNA were measured by qRT‒PCR. (**F**-**K**) Lp-PLA2, IL-6, IL-1β, and TNF-α concentrations were determined by ELISAs. Parametric data were analysed by paired t tests and are displayed as the mean ± SD. *N* = 3, * *p* < 0.05, ** *p* < 0.01, *** *p* < 0.001, **** *p* < 0.0001

To investigate possible alterations in miR-494-3p expression in LPS-stimulated RAW264.7 cells, we conducted qRT‒PCR analysis. The results demonstrated the upregulation of miR-494-3p expression following LPS stimulation (Fig. 3A). We transfected the miR-494-3p mimic and inhibitor into LPS-stimulated RAW264.7 cells and compared those cells with control cells to determine whether miR-494-3p regulates the inflammatory response. A subsequent analysis was conducted using qRT‒PCR to evaluate the efficiency of the transfection process and determine the relative expression of IL-1β, IL-6, and TNF-α (Fig. 3B). The overexpression of miR-494-3p in RAW264.7 cells treated with LPS significantly decreased the expression levels of IL-1β, IL-6, and TNF-α. To validate this discovery, we used a miR-494-3p inhibitor to suppress its expression, resulting in an increase in inflammatory factors in RAW264.7 cells (Fig. 3C-E); The ELISA results supported the abovementioned findings (Fig. 3F-H). Based on the results of these experiments, miR-494-3p mitigates the inflammatory response in LPS-stimulated RAW264.7 cells.

### Impact of mir-494-3p on LPS-stimulated RAW264.7 cell viability and proliferation

**Fig. 4:**
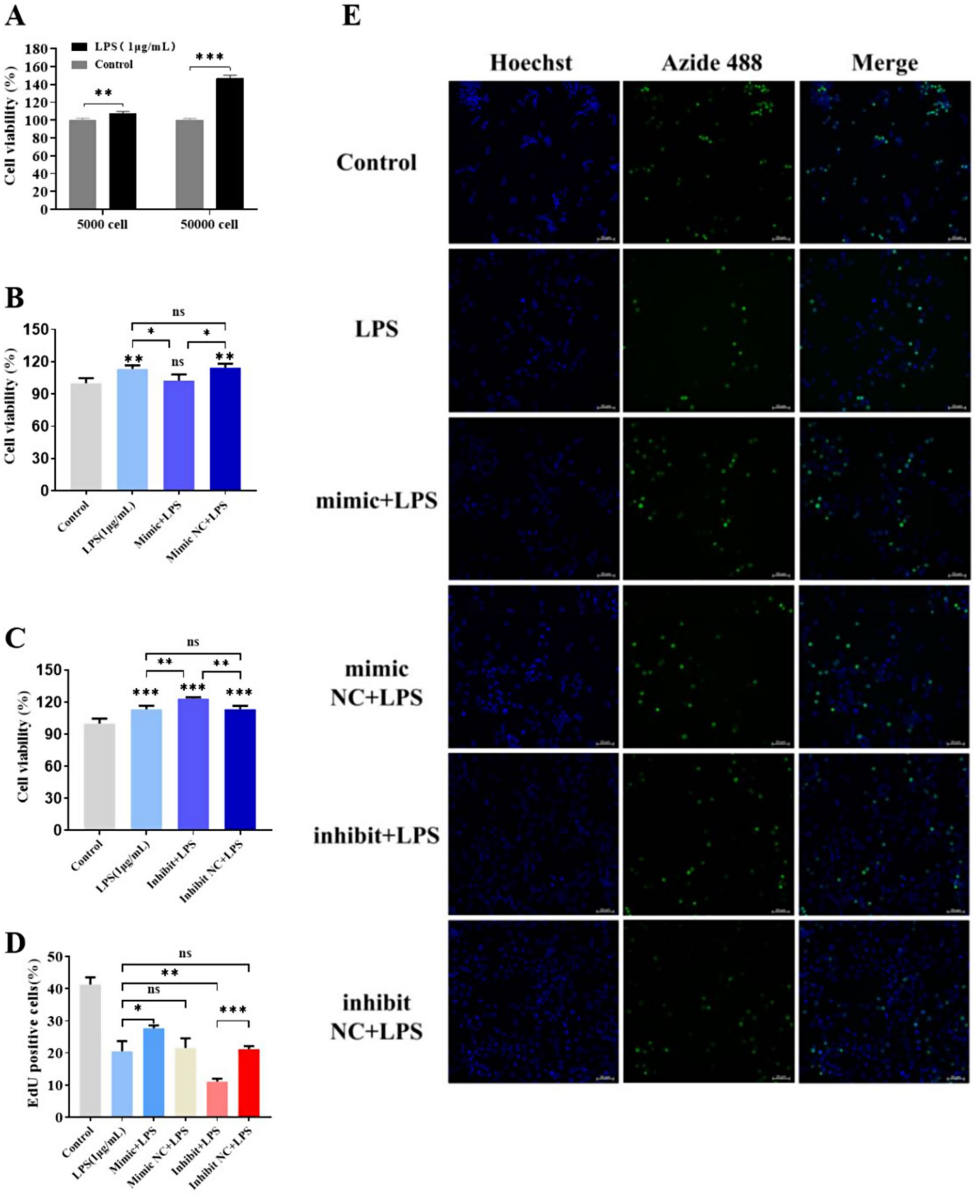
CCK8 assay and EdU staining results. (**A**) CCK-8 assays comparing the control group and LPS-treated group. (**B**) CCK-8 assays after overexpressing miR-494-3p. (**C**) CCK-8 assays after downregulating miR-494-3p expression. (**D** and **E**) EdU staining results for each group. Green, EdU-positive cells; blue, Hoechst 33,342 (nucleus). Scale bar, 50 μm. Parametric data were analysed by paired t tests and are displayed as the mean ± SD. *N* = 3, * *p* < 0.05, ** *p* < 0.01, *** *p* < 0.001

To examine the impact of miR-494-3p on the viability of LPS-stimulated RAW264.7 cells, a CCK-8 assay was performed. Based on the findings, LPS stimulation enhanced cellular metabolism, whereas the overexpression of miR-494-3p inhibited this enhancement; conversely, the downregulation of miR-494-3p expression further enhanced cellular metabolism (Fig. 4A-C). EdU cell proliferation experiments were conducted to investigate the effect of miR-494-3p on cell proliferation. LPS stimulation inhibited cellular proliferation, which was mitigated by the upregulation of miR-494-3p expression and exacerbated by the downregulation of miR-494-3p expression (Fig. 4D-E). The data presented herein demonstrate that miR-494-3p inhibits the LPS-induced activation of RAW264.7 cells while simultaneously mitigating the LPS-induced inhibition of proliferation.

### Protective effect of mir-494-3p in LPS-stimulated RAW264.7 cells

**Fig. 5:**
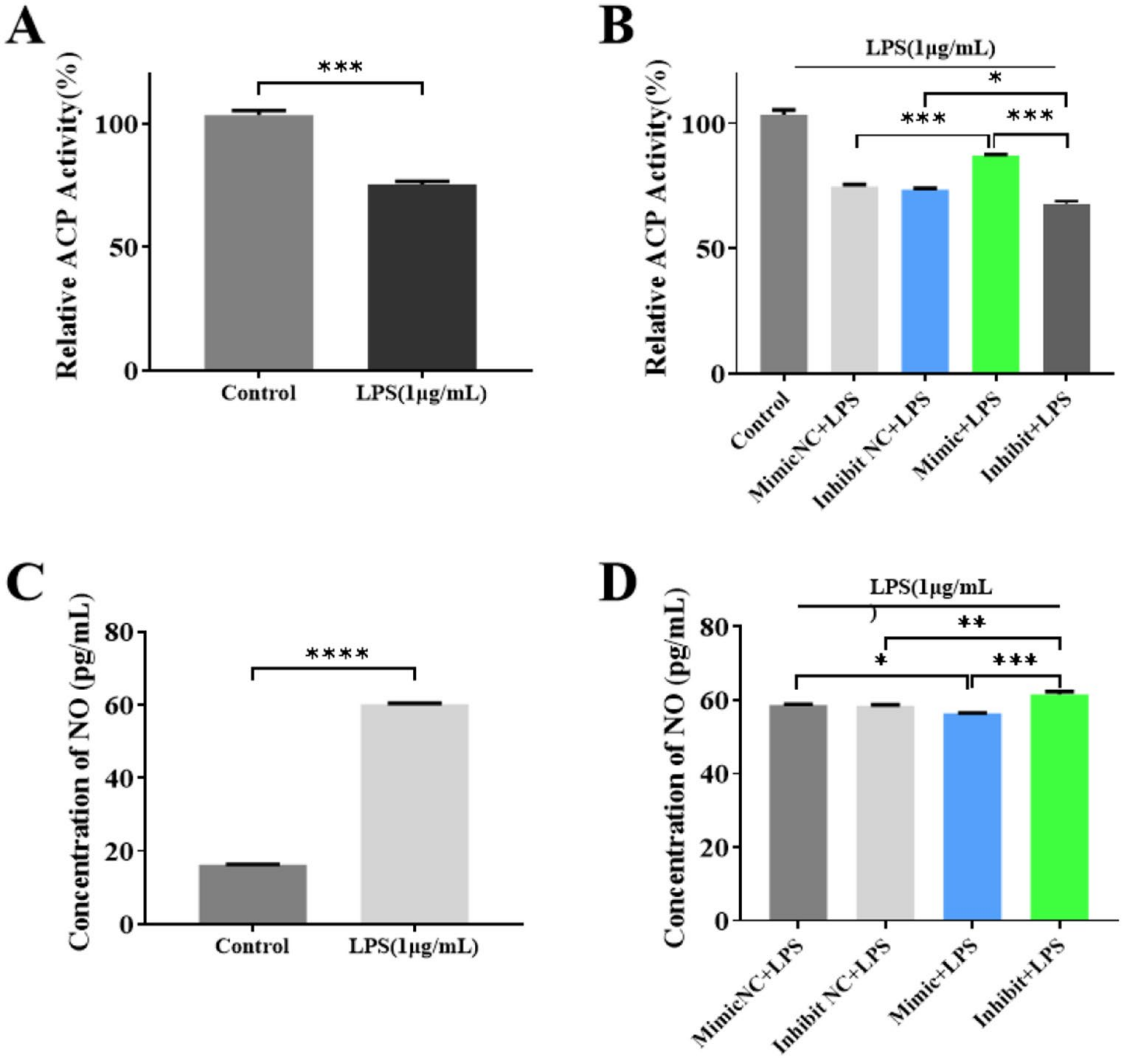
Effects of miR-494-3p on intracellular ACP activity and the concentration of NO in LPS-stimulated RAW264.7 cells. (**A** and **B**) Effects of LPS on the activity of ACP in RAW264.7 cells. (**C** and **D**) NO secretion by RAW264.7 cells. Parametric data were analysed by paired t tests and are displayed as the mean ± SD. *N* = 3, * *p* < 0.05, ** *p* < 0.01, *** *p* < 0.001. **** *p* < 0.0001

We found that LPS stimulation resulted in a decrease in intracellular ACP activity (Fig. 5A). The overexpression of miR-494-3p alleviated the LPS-induced decrease in ACP activity in RAW264.7 cells; the knockdown of miR-494-3p made this LPS-induced decrease more pronounced (Fig. 5B). In addition, LPS stimulation significantly increased NO release from RAW264.7 cells (Fig. 5C), while the overexpression of miR-494-3p reduced NO release, and the knockdown of miR-494-3p increased this release (Fig. 5D). In conclusion, these findings suggest that miR-494-3p protects against LPS-induced damage in RAW264.7 cells.

## Discussion

Our team investigated the potential roles of Lp-PLA2-targeting microRNAs (miRNAs) in LPS-induced inflammation in RAW264.7 cells. Previous studies have demonstrated that RAW264.7 cells can be stimulated with LPS to create a cellular model of sepsis [19]. LPS can induce M1-like macrophages, and the upregulation of TNF-α [20] and iNOS [21] are biomarkers of M1 activation [22, 23]. As demonstrated by our results, RAW264.7 cells stimulated with LPS released more TNF-α and NO, indicating the successful establishment of an inflammation model.

Lp-PLA2 has attracted much attention in recent years [24, 25]; when released, it is oxidized and cleaved into biologically active compounds that are thought to have inflammatory properties [26, 27]. In addition, there has been an increase in research focusing on miRNAs as key components of innate immunity [11, 28–30]. Exosomal miR-30d-5p from polymorphonuclear neutrophils (PMNs) promotes sepsis-related acute lung injury (ALI) by activating M1 macrophage polarization and inducing macrophage pyroptosis through NF-B signalling [31]. In our study, Lp-PLA2 was confirmed to be a target of microRNA-494-3p via dual-luciferase reporter assays, and miR-494-3p inhibited the release of Lp-PLA2.

Sepsis is a life-threatening inflammatory response that damages organs. Inhibiting the inflammatory response is an effective way to treat sepsis. To investigate the inhibition of inflammatory responses, it is crucial to understand what specific cytokines are released and which signalling pathways are activated when the immune system is stimulated with LPS [32–34]. The results of this study showed that miR-494-3p overexpression reduced the release of LPS-induced inflammatory factors in RAW264.7 cells, weakened the effect of LPS on RAW264.7 cell function and played an anti-inflammatory role. Therefore, targeting miR-494-3p may be a promising therapeutic strategy for the management of sepsis.

Although the present study yielded valuable results regarding the role of miR-494-3P in sepsis, there are some notable limitations. The findings were only verified at the cellular level; that is, in vivo animal experiments were not conducted, and clinical data were not analysed. In addition, there is a lack of studies on the role of miR-494-3p in signalling pathways associated with inflammation. In addition to in vivo animal experiments, a focus of future investigations of miR-494-3p will be to elucidate the precise mechanism by which miR-494-3p inhibits inflammation through Lp-PLA2.

## Conclusion

There have been many advances in epidemiological research, risk factor identification, pathophysiology understanding, and prognosis assessments for patients with sepsis, all of which have greatly contributed to our understanding of this condition and improved our ability to prevent, detect, and treat it effectively. However, sepsis remains a substantial global health concern requiring further investigation to assist clinicians in early recognition, diagnosis, and treatment. Building upon our previous findings of elevated levels of Lp-PLA2 in the serum of sepsis patients, the results of this study demonstrated that miR-494-3p mitigates LPS-induced inflammation in RAW264.7 cells by suppressing the secretion of Lp-PLA2, thereby conferring a certain degree of cellular protection.
